# Supplementary material for: An assessment of the fixin tplo jig to generate effective compression using a transverse fracture model
Source: PLoS One. 2023 Oct 13;18(10):e0286937. doi: 10.1371/journal.pone.0286937 (PMC10575488; doi:10.1371/journal.pone.0286937)
Supplement: S1 File — (PDF) [file pone.0286937.s001.pdf]

MANUAL

TESNSIONING

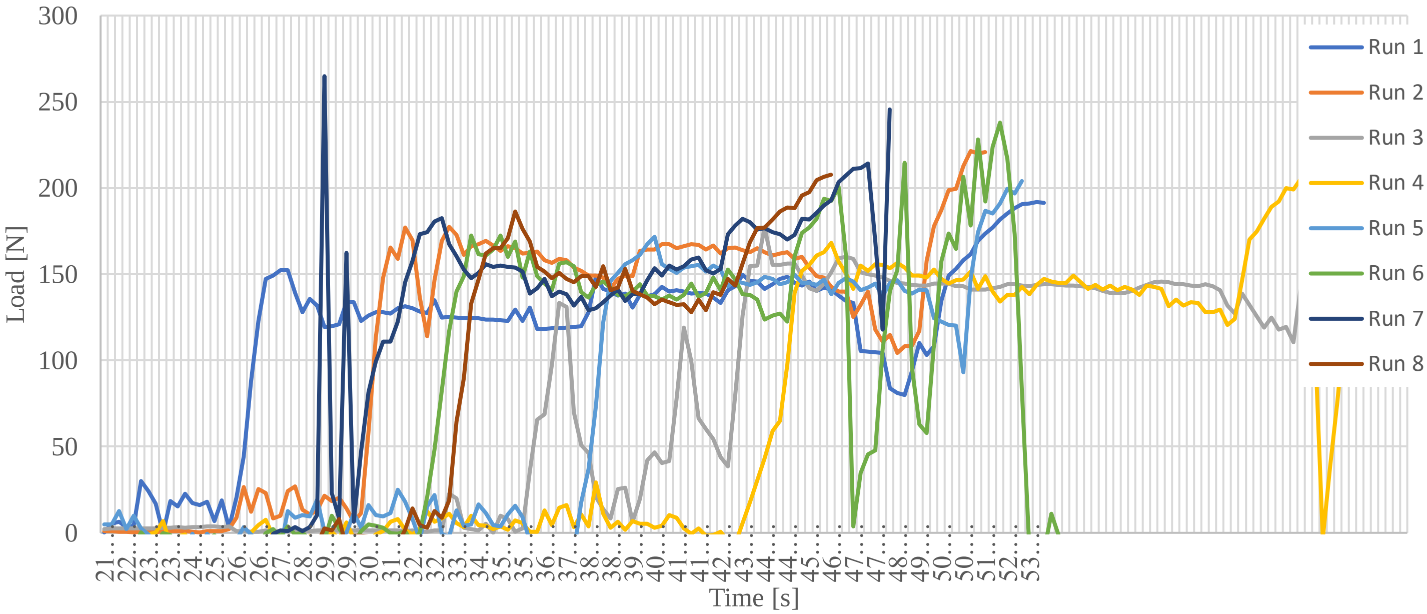

Fig. 15. The load measured by the load cell as the needle holders coiled the wire for each of the eight runs.

AO TENSIONING DEVICE

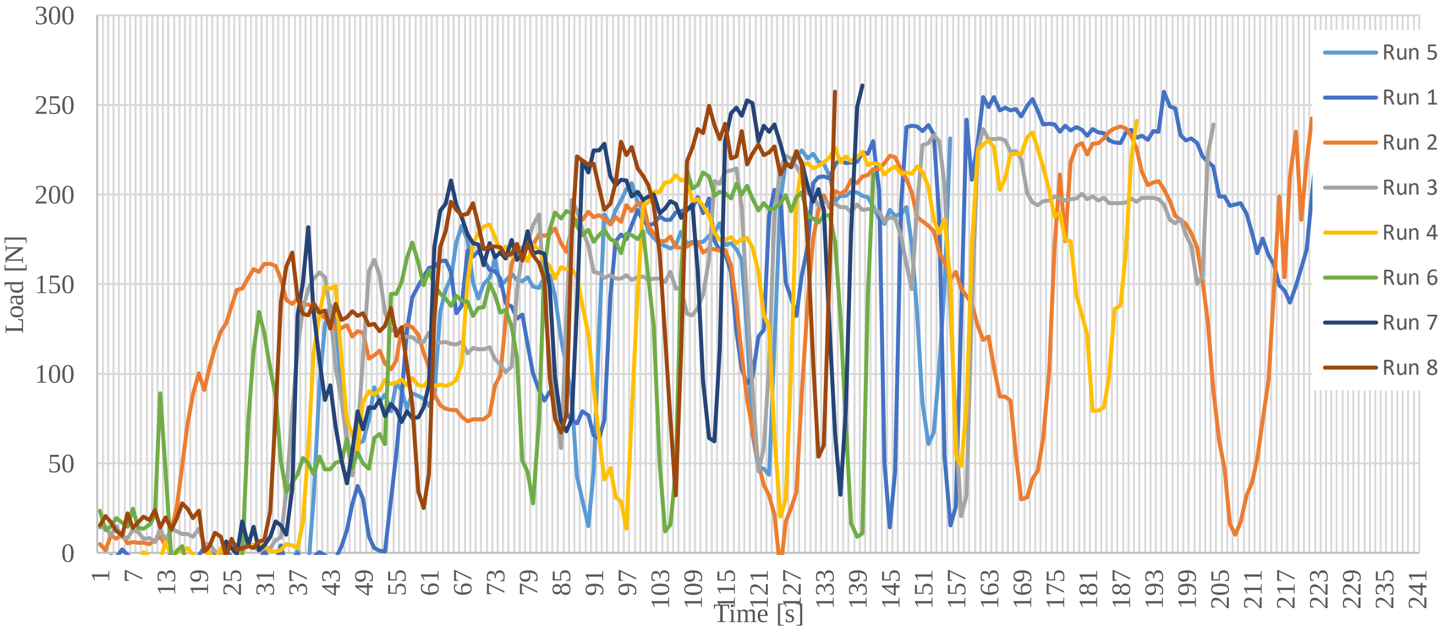

Fig. 16. The load measured by the load cell as the AO tensioning device coiled the wire for each of the eight runs.

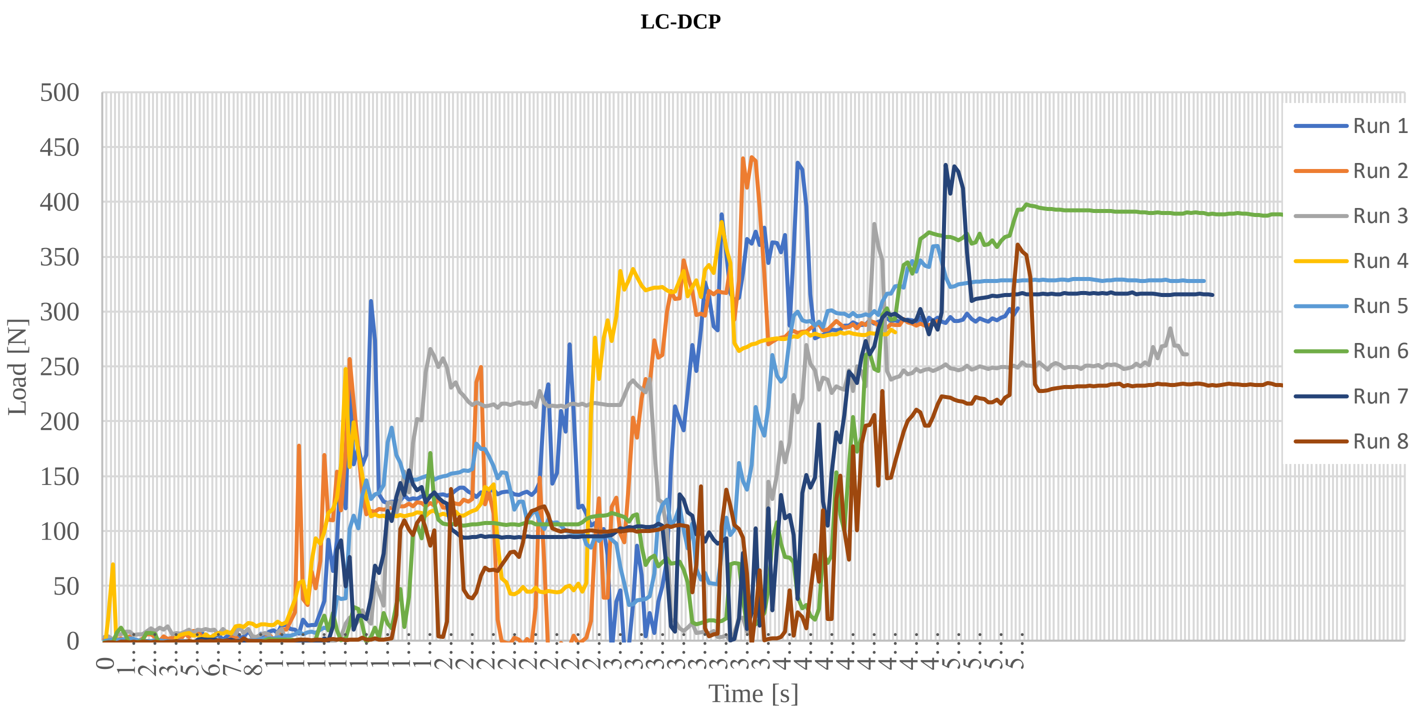

Fig. 17. The load measured by the load cell during LC-DCP
