# Supplementary material for: An assessment of the fixin tplo jig to generate effective compression using a transverse fracture model
Source: PLoS One. 2023 Oct 13;18(10):e0286937. doi: 10.1371/journal.pone.0286937 (PMC10575488; doi:10.1371/journal.pone.0286937)
Supplement: S2 File — (PDF) [file pone.0286937.s002.pdf]

One-Way ANOVA Calculator, Including Tukey HSD

Success!

Explanation of results

The output of this calculator is pretty straightforward. The values of *f* and *p* appear at the bottom of the page. If the text is blue, your result is significant; if it's red, it's not. The only thing that might catch you out is the way that we've rounded the data. The data you see in the tables below, which provide details about the calculation, has been rounded. However, we did not round when actually calculating the values of *f* and *p*. This means that if you try to calculate these values on the basis of the summary data provided here, you're likely going to end up with a slightly different - and less accurate - result.

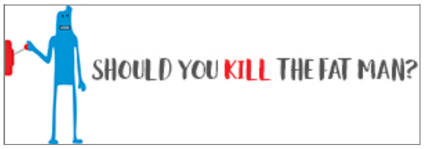

Treatment 1	Treatment 2	Treatment 3	Treatment 4	Treatment 5
133.61	238.01	290.63	189.04	133.25
157.68	230.98	285.42	177.62	127.58
145.48	197.06	251.16	150.62	215.65
147.21	216.79	276.87	163.56	115.18
148.33	200.04	328.21	152.92	154.76
147.71	197.27	389.23	147.42	106.29
149.93	227.12	315.99	169.29	94.81
155.65	226.14	233.03	174.82	100.98

Summary of Data

	Treatments					
	1	2	3	4	5	Total
N	8	8	8			40
∑X	1185.6	1733.41	2370.54			7663.34
Mean	148.2	216.6763	296.3175			191.584
∑X2	176075.7894	377485.3167	719043.1738			1641903.5352
Std.Dev.	7.269	16.4601	48.713			66.7437

Result Details

Source	***SS***	***df***	***MS***	

Between-treatments	142525.9153	4	35631.4788	*F* = 39.96081
Within-treatments	31208.121	35	891.6606	
Total	173734.0363	39		

The *f*-ratio value is 39.96081. The *p*-value is < .00001. The result is significant at $p < .05$.

Post Hoc Tukey HSD (beta)

The Tukey's HSD (honestly significant difference) procedure facilitates pairwise comparisons within your ANOVA data. The F statistic (above) tells you whether there is an overall difference between your sample means. Tukey's HSD test allows you to determine between which of the various pairs of means - if any of them - there is a signficant difference.

A couple of things to note. First, a blue value for Q (below) indicates a significant result. Second, it's worth bearing in mind that there is some disagreement about whether Tukey's HSD is appropriate if the F-ratio score has not reached significance.

Pairwise Comparisons		$HSD_{.05}$ = 42.9251<br>$HSD_{.01}$ = 52.5798	$Q_{.05}$ = 4.0659 $Q_{.01}$ = 4.9804
**T_1:T_2**	M_1 = 148.20<br>M_2 = 216.68	68.48	Q = 6.49 (*p* = .00050)
**T_1:T_3**	M_1 = 148.20<br>M_3 = 296.32	148.12	Q = 14.03 (*p* = .00000)
**T_1:T_4**	M_1 = 148.20<br>M_4 = 165.66	17.46	Q = 1.65 (*p* = .76832)
**T_1:T_5**	M_1 = 148.20<br>M_5 = 131.06	17.14	Q = 1.62 (*p* = .78014)
**T_2:T_3**	M_2 = 216.68<br>M_3 = 296.32	79.64	Q = 7.54 (*p* = .00005)
**T_2:T_4**	M_2 = 216.68<br>M_4 = 165.66	51.01	Q = 4.83 (*p* = .01316)
**T_2:T_5**	M_2 = 216.68<br>M_5 = 131.06	85.61	Q = 8.11 (*p* = .00002)
**T_3:T_4**	M_3 = 296.32<br>M_4 = 165.66	130.66	Q = 12.38 (*p* = .00000)
**T_3:T_5**	M_3 = 296.32<br>M_5 = 131.06	165.25	Q = 15.65 (*p* = .00000)
**T_4:T_5**	M_4 = 165.66<br>M_5 = 131.06	34.60	Q = 3.28 (*p* = .16353)

Calculate Reset

 ©2021 Jeremy Stangroom
