# Supplementary material for: An assessment of the fixin tplo jig to generate effective compression using a transverse fracture model
Source: PLoS One. 2023 Oct 13;18(10):e0286937. doi: 10.1371/journal.pone.0286937 (PMC10575488; doi:10.1371/journal.pone.0286937)
Supplement: S3 File — (PDF) [file pone.0286937.s003.pdf]

Group a needle holder

	Load at Quarter Turn [N]	Max Load [N]	relative sta
	133.61	191.86	
	157.68	221.43	
	145.48	220.73	
	147.21	214.56	
	148.33	204.03	
	147.71	237.91	
	149.93	245.58	
	155.65	207.71	
Average	148.2	217.97625	
STD	7.269008578	17.61677767	
RSd	4.904864088	8.081971163	

Group wire tensoning device

	Load at 1/2 Rotation [N]	Load at 1 Rotation [N]	ad at 1-1/2 Rotations	t 2 Rotatic
	156.09	189.04	218.52	238.01
	133.12	177.62	210.06	230.98
	118.72	150.62	192.58	197.06
	94.02	163.56	190.79	216.79
	86.94	152.92	182.17	200.04
	50.96	147.42	179.66	197.27
	79.46	169.29	198.35	227.12
	131.56	174.82	212.81	226.14
Average		165.66125		216.6763
STD		14.69871368		16.46007
RSd		8.872753093	#DIV/0!	7.596621

Group D

Load after Upper Compression Screw Tig	r Lower Compression Screw Tight	Max Load [N]
133.25	290.63	435.78
127.58	285.42	440.79
215.65	251.16	379.93
115.18	276.87	381.68
154.76	328.21	359.84
106.29	389.23	397.78

	94.81	315.99	433.79
	100.98	233.03	361.13
Average	131.0625	296.3175	398.84
STD	39.31313984	48.71298939	33.68359075
RSd	29.99571948	16.43945747	8.445389316

