# Supplementary material for: An assessment of the fixin tplo jig to generate effective compression using a transverse fracture model
Source: PLoS One. 2023 Oct 13;18(10):e0286937. doi: 10.1371/journal.pone.0286937 (PMC10575488; doi:10.1371/journal.pone.0286937)
Supplement: Attachment — Submitted filename: Response to Reviewers 2 jig project.docx [file pone.0286937.s004.docx]

July 27, 2023

**Dr. Mohamed El-Sayed Abdel-Wanis**

**Academic Editor PLOS ONE**

Dear Editor,

I believe that revisor two did not ask any questions.

Sincerely,

**Cassio Ferrigno, DVM, M.S. PhD**

**Assistant Professor of Orthopedic Surgery**

SMALL ANIMAL CLINICAL SCIENCES
