# Supplementary material for: An assessment of the fixin tplo jig to generate effective compression using a transverse fracture model
Source: PLoS One. 2023 Oct 13;18(10):e0286937. doi: 10.1371/journal.pone.0286937 (PMC10575488; doi:10.1371/journal.pone.0286937)
Supplement: Attachment — Submitted filename: second revision Response to editor 1 jig project.docx [file pone.0286937.s005.docx]

September 26, 2023, 2023

**Dr. Mohamed El-Sayed Abdel-Wanis**

**Academic Editor PLOS ONE**

Dear Editor,

We have included all the relevant information discussed with the first reviewer in the text as requested.

We will indicate where in the text new information was added or discussed previously.

*“The 3D printed APS plastic was selected to simulate bone. Is it similar in its biomechanical properties to the bone? If so provide evidence. There must be a rationale why natural bones weren't used.”*

This information was added in the lines 243-246

*“As an LCDCP plate utility is clear, it is not clear if the TPLO jig practical in clinical scenarios? In which exactly? Please provide an explanation, as not all of the readership of this journal come from the Veterinarian community.”*

The use of neutral locking plates in veterinary orthopedics is becoming increasingly popular. However, many new generation implants lack the compress hole in the plate. Thus, using the TPLO jig has the potential to compress the fracture gap using a neutral implant. In some fractures, if a stronger compression is desired, the jig could be used. Additionally, in a MIPO scenario where achieving good compression in the fracture gap is not always possible, this concept could also be utilized.

The information was added in lines 309 to 313.

*“Line 256-262: A fair point, but the correlation to practical real life clinical decision is missing. In which wire will the authors recommend to use in fracture surgery? If other than 18 gauge, why was this wire selected for their model? If not, what is their explanation?”*

The information was previous discussed in lines 286 -295

Sincerely,

**Cassio Ferrigno, DVM, M.S. PhD**

**Assistant Professor of Orthopedic Surgery**

SMALL ANIMAL CLINICAL SCIENCES
